# E8-LWDS: Factorial Structure and Psychometric Properties of the Lebanese Waterpipe Dependence Scale-11 in 1490 Egyptian Waterpipe Tobacco Smokers—A Critical Approach

**DOI:** 10.3390/ijerph18136741

**Published:** 2021-06-23

**Authors:** Aya Mostafa, Nashwa Ismail

**Affiliations:** Department of Community, Environmental, and Occupational Medicine, Faculty of Medicine, Ain Shams University, Cairo P.O. Box 11566, Egypt; nashwa_aly@med.asu.edu.eg

**Keywords:** waterpipe tobacco smoking, dependence, factor analysis, validity, reliability, psychometric properties, LWDS, Egypt

## Abstract

Introduction: There is no universal scale for assessing waterpipe tobacco (WT) dependence. We examined the factorial structure and psychometric properties of the Waterpipe Dependence Scale-11 (LWDS-11) among Egyptian WT smokers. Methods: We administered the LWDS-11 during face-interview questionnaires in two cross-sectional surveys among 1490 current WT smokers recruited via purposive quota sampling. Exploratory factor analysis was performed on half of the sample. Confirmatory factor analysis of the resulting model was done using structural equation modelling on the other half. Scale reliability was examined. We assessed convergent construct validity using regression models to examine the association between the adapted dependence scale and factors conceptually expected to be associated with WT dependence. Results: Exploratory factor analysis of the scale yielded eight items (E8-LWDS) supporting a three-factor structure: physical dependence (three items); psychological dependence (three items); and psychological craving (two items). Cronbach’s α were 0.635 for the total scale and 0.823, 0.654, and 0.785 for the three subscales. E8-LWDS was confirmed to have good model fit (comparative fit index = 0.995; root mean squared error of approximation = 0.027). E8-LWDS was independently associated with daily WT smoking, rural residence, being a skilled worker, non-exclusive WTS, smoking ≥ eight WT hagars/day, and measures of perceived behavioral control (self-reported addiction to WT, perceived ability to quit, and previous quit attempts). Conclusion: E8-LWDS showed adequate psychometric properties among this sample of Egyptian current WT smokers, which makes it appropriate for use by researchers and practitioners. Adding items related to perceived behavioral control might enhance the scale robustness.

## 1. Introduction

Waterpipe tobacco smoking (WTS) is a rapidly evolving global public health concern because of its associated toxicant inhalation, disease risk, and dependence [1]. Misperception of WTS harm and nicotine content has partly contributed to its increasing popularity worldwide [2,3,4]. WTS rates in youth reached 11.4%, 22.7% and 37.2% in some reports from the United States, Latvia, and Lebanon, respectively [5]. In the Eastern Mediterranean region, WTS rates are the highest and have surpassed cigarette smoking in adolescents [5,6,7].

WTS-associated diseases are well-documented (cancers; respiratory, cardio-vascular, and periodontal diseases; and obstetrical complications) [8]. Similar to cigarette smoking, WTS exposes smokers to dangerous toxicants (carbon monoxide, volatile aldehydes, polycyclic aromatic hydrocarbons, and nitrosamines), heavy metals (cadmium and lead), and the dependence-producing drug, nicotine [1]. Moreover, WTS potentiates cigarette smoking [9]; thus, increasing the likelihood of nicotine dependence through multiple tobacco product use.

There is compiling evidence that WTS supports nicotine dependence [10]. Dependence is a multidimensional matter; thus, characterizing features unique to waterpipe tobacco (WT) dependence is important in informing cessation efforts. For instance, initiating WTS at a younger age, smoking WT daily, transitioning from a social to an individual WTS pattern, and lower perceived WTS addictiveness and lower self-efficacy were found to be associated with WT dependence [11,12,13,14,15,16,17,18,19,20]. However, WT smokers may not be aware of such features. Egyptian WT smokers exhibit many similar characteristics of nicotine dependence like those attributed to cigarette smokers [14]. A quarter of Egyptian WT smokers self-reported addiction to WTS and 28.1% were uncertain whether they were WT dependent [20]. In the absence of waterpipe-specific measures of tobacco dependence, WTS addictiveness may be currently underestimated [21]. Therefore, a tool for screening smokers to identify dependent subjects who need greater assistance to control their WTS behavior is currently needed in clinical situations.

To address this gap, Salamah et al. proposed the Lebanese Waterpipe Dependence Scale (LWDS-11) [22]. Its 11 items represent four domains: “physiological nicotine dependence”, “negative reinforcement”, “psychological craving”, and “positive reinforcement” [22]. The scale was evaluated in Jordan [23], Turkey [24], Iran [25], and the UK [26] for reliability, validity, and psychometric properties. However, these studies were confined to certain waterpipe smoker groups (small numbers; selective age or gender; café WTS, non-current WTS, or exclusive WTS) [22,23,24,25,26] and revealed some differences from the original scale (Supplementary Table S1) [23,24,25,26].

To date, this scale has not been evaluated in Egypt, one of the largest WT markets [27], where treatment of tobacco-related diseases costs US$616 million annually and 170,000 tobacco-attributable deaths occur each year [28]. Egyptian adolescent girls (4.1%), boys (7.2%), and university students (25.4%) report high WTS rates relative to adult women (0.1%) and men (8.7%) [29,30,31]. Most adult WT smokers smoke it daily and at home [31], and are rurally located [32]. Multiple nicotine/tobacco product use among WT smokers is not uncommon [7]. Thus, it would be particularly valuable to address this unique Egyptian WTS context and assess the LWDS-11 among a more diverse population of WT smokers. To provide public health researchers and practitioners with a validated screening tool for WT dependence, we examined the factorial structure and psychometric properties of LWDS-11 among a large population of current Egyptian WT smokers using exploratory and confirmatory factor analysis.

## 2. Methods

Data for the current study was collected during conducting a larger study on WT smokers in Egypt. Details of the larger study design, sampling, participants, instrument, and procedures have been described elsewhere [4,7,20]. It comprised two identical cross-sectional surveys. In 2015–2017, individuals ≥18 years old residing in rural and urban areas were recruited using purposive quota sampling. After obtaining informed consent, participants completed a face-to-face interview survey at cafes, households, workplaces, and universities. In the current study, survey data on the 1490 current WT smokers, including participants’ background characteristics; WT dependence using the LWDS-11; and WTS behaviour, perceived harm and perceived behavioural control of WTS—being measures conceptually expected to be associated with dependence—were analysed.

### 2.1. Measures

#### 2.1.1. Background Characteristics

Data on participants’ background characteristics included age, gender, urban/rural residence, educational level, occupation, marital status, and self-reported household exposure to second-hand smoke.

#### 2.1.2. Waterpipe Tobacco Dependence Using LWDS-11

The eleven items of the LWDS-11 that were used in this study to assess current WT smokers’ dependence on WT use, their response options, and scoring were published earlier by Salama et al. [22]. As previously described in detail [4], the study instrument—including the LWDS-11 items—were assessed for face and content validity, were pilot tested, and were administered in Egyptian colloquial Arabic. Each of the LWDS-11 items scored 0–3 points; the total score of the original LWDS-11 ranged 0–33 points.

#### 2.1.3. Waterpipe Tobacco Use Behavior, Perceived Harm, and Perceived Behavioral Control

To examine convergent construct validity of the adapted scale among this study population, measures that were hypothesized to be associated with WT dependence, such as younger age at WTS initiation, higher frequency and intensity of WTS, smoking alone and at home, less perceived harm of WTS compared with cigarette smoking, self-reported addiction to WTS, lower self-efficacy, and fewer quit attempts were identified from previous literature [10,11,12,13,14,15,16,17,18,19,20,22,23]. Hence, data on participants’ WTS behavior, perceived harm, and perceived behavioral control of WTS were included in this analysis.

Waterpipe tobacco use behavior: Participants were considered current WT smokers if they reported any WTS in the 30 days before the survey. To identify multiple nicotine/tobacco product use, WT smokers were asked whether they had smoked cigarettes or used electronic nicotine delivery systems (ENDS) in the 30 days before the survey. Here forth, non-exclusive WTS refers to WT and/or cigarette smoking and/or ENDS use. In addition, the following data on WTS behavior were obtained: age at WTS initiation (years), WTS frequency (monthly/weekly/daily); WTS intensity (number of WT hagar/portions smoked a day); usual place where participants smoked the waterpipe (at home or other places); whether they usually smoked the waterpipe alone or in the company of others; usual source of WT (self-purchase or other sources); and average monthly spending on WTS—prices were reported in Egyptian pounds and converted to US$.

Perceived harm of WTS: participants were asked how harmful they thought WTS is as compared with cigarettes (more harmful, about the same, less harmful, do not know); how much nicotine was in WTS compared with cigarettes (more nicotine, about the same, less nicotine, do not know); and how often they worried about the health hazards of WTS (never, sometimes, often).

Perceived behavioral control of WTS: Participants were asked about their perceived behavioral control of WTS: whether they considered themselves addicted (self-reported addiction) to WTS (yes, no, do not know); if they were confident they could quit WTS (perceived self-efficacy) any time they wanted (yes/no); and whether they had attempted to quit WTS in the past (yes/no).

### 2.2. Ethical Considerations

This study was approved by the Ethical Review Board of Faculty of Medicine, Ain Shams University (FMASU R 10/2015 and 10a/2016). All participants provided informed consent. Participation was voluntary, data were collected anonymously using serial study IDs, and data confidentiality was ensured.

### 2.3. Analysis

Exploratory factor analysis (EFA) was performed on one half of the sample and confirmatory factor analysis (CFA) on the other half. The sample was divided randomly using the automatic function in SPSS version 25. The 11 items from the LWDS-11 were introduced into the EFA. EFA was performed on the first dataset (*n* = 747) using principal-component analysis. First, we checked the suitability of the data for factor analysis by performing Kaiser–Meyer–Olkin (KMO) measure of sampling adequacy and Bartlett’s χ^2^ test of sphericity. The number of factors was inspected visually using the scree plot, and was evaluated by examining the Kaiser Criterion of eigenvalues ≥1 [33], and the proportion of variance explained by each factor to identify the ideal number of latent factors [33]. Items were reserved in the factor structure if the strength of item loadings on factors was ≥0.4 [33]. Reliability was assessed by calculating Cronbach α to examine the internal consistency of the scale, factors and items. Internal consistency values >0.7 are considered good scale reliability but may be reduced to >0.6 in exploratory studies [33]. Principal-component analysis was repeated using Varimax rotation [33]. The resulting latent factors from the EFA were assessed conceptually. Because multiple nicotine/tobacco product use among WT smokers is not uncommon [7] and cessation services generally target all WT smokers not only exclusive WT smokers, we also conducted EFA of the dependence scale following the same steps described above in samples of exclusive WT smokers (*n* = 777) and non-exclusive smokers (*n* = 713) to assess whether the resultant model and factor structure was different between these WT smoker groups.

Then CFA was performed on the second dataset (*n* = 743) using structural equation modeling (IBM AMOS version 16) to examine the fit of the model resultant from the EFA. There were no missing observations in the variables included in the CFA. The maximum likelihood estimation method was applied. Covariance was included among each pair of factors in the model. Unstandardized and standardized estimates of regression weights as well as covariances and correlations between variables were obtained, and modification indices were examined. As the data were non-normally distributed, we performed Bullen-Stine bootstrapping to test the null hypothesis that the model is correct. We report indices of model fit [33]: the Tucker-Lewis index (TLI), the comparative fit index (CFI), the root mean square error approximation (RMSEA), and the *p*-value for a close fit (PCLOSE). A good model fit was considered if the CFI >0.90, TLI > 0.90, RMSEA < 0.05 [33]. The ratio Chi-square on degrees of freedom (χ^2^/df < 2.00) was also assessed [34].

We conducted bivariable and multivariable analyses to examine convergent construct validity between the dependence scale (E8-LWDS) that was obtained from EFA and CFA (dependent variable) and its associations with the other survey measures that were hypothesized to be associated with WT dependence (independent variables: WTS behavior, perceived harm and perceived behavioral control of WTS). The dependent variable was further categorized into three categories (low, moderate, and high dependence) using tertiles as proposed by Primack et al. [23]. In the bivariable analysis, the dependent variable was entered as a categorical variable; associations with the independent variables were examined using the chi-squared test for categorical variables and the analysis of variance test (ANOVA) for continuous variables. In the multivariable analysis, the dependent variable was entered as a continuous variable into a generalized linear model with all independent variables, which were dichotomized for the purpose of this analysis: background characteristics (age, gender, residence, educational level, occupation, marital status, and household exposure to second-hand smoke, current cigarette smoking); WTS behavior (age at WTS initiation, frequency and intensity of WTS, usual place where participants smoked the waterpipe, usually smoked the waterpipe alone or in the company of others, usual source of waterpipe tobacco, average monthly spending on WTS); perceived harm of WTS (perceived harm of WTS compared with cigarettes, perceived nicotine content in WTS compared with cigarettes, worry about the health hazards of WTS); perceived behavioral control of WTS (self-reported addiction to WTS, perceived self-efficacy to quit WTS, and previous attempts to quit WTS). Besides testing for convergent construct validity, this analysis was carried out to identify other variables that have potential to be included in future scales. Beta coefficients and 95% confidence intervals (CI) are reported. Furthermore, we assessed the ability of the adapted dependence scale to discriminate between different levels of dependence among current WT smokers by the intensity and frequency of WTS using median nonparametric tests: non heavy smokers (<8 hagars/day or <7 waterpipes/week) versus heavy smokers (≥8 hagars/day or ≥7 waterpipes/week) [14,24]. These two measures generally carry adequate face validity to discriminate between groups of smokers [11,14,24]. For all analyses, a *p*-value ≤0.05 was used as the threshold for statistical significance.

## 3. Results

### 3.1. General Characteristics of the Study Population (N = 1490)

Participants’ age ranged from 18–87 (median = 35.0; IQR:23.0–46.0). Females represented 8.7% of WT smokers in this sample. Almost half (47.9%) of WT smokers reported non-exclusive WTS. Other participant characteristics are presented in Table 1.

### 3.2. Exploratory Factor Analysis (n = 747)

EFA was done on the first half of the dataset. First, principal components analysis was done including all eleven items of the LWDS using a cut-off point of 0.4 for factor loadings. The data were found to be suitable for EFA; KMO was greater than 0.5 (0.685) and Bartlett’s test of sphericity was significant (<0.001). By examining the scree plot, a three-factor solution was suggested. Three factors yielded eigenvalues >1.0. A varimax rotation type of factor analysis was suitable as the factors were orthogonal. The EFA was repeated with varimax rotation, where two items that had factor loadings < 0.4 were suppressed: “percent of income you would spend on waterpipe smoking” (0.267) and “do you smoke waterpipe alone” (0.249). Next, reliability tests were done for the items that loaded on each factor and for each of the three factors. The reliability of Factor 1 was initially low (α = 0.213); removing the last item that loaded on this factor “do you smoke to please others” improved the reliability of Factor 1 (α = 0.823). EFA and reliability tests were repeated on the remaining eight items. Factor loadings of the eight items on the three factors are shown in Table 2. The total eight-item scale reliability was α = 0.635. Three items loaded on Factor 1 (α = 0.823), three items loaded on Factor 2 (α = 0.654), and two items loaded on Factor 3 (α = 0.785). This eight-item model explained 75.02% of the variance in the sample. Conceptually, the three items loading on Factor 1 were related to physical dependence; therefore, Factor 1 was labelled “physical dependence.” The three items loading on Factor 2 were related to WTS psychological aspects including relaxation, improving morale and pleasure; thus, Factor 2 was labelled “psychological dependence.” Finally, the two items loading on Factor 3 were related to difficulties facing WT smokers in controlling their impulses to smoke, even in situations where they should; hence, Factor 3 was labelled “psychological craving” (Table 2).

We further examined if there was a difference in the application of LWDS-11 on exclusive and non-exclusive WT smokers. EFA was carried out following the previous steps on a sample of exclusive (*n* = 777) and on a sample of non-exclusive WT smokers (*n* = 713). Similar models were obtained, except that the total Cronbach’s alpha for the model in the non-exclusive WT smoker group slightly decreased (0.600). The eight-item model explained 70.79% of the variance in the sample of exclusive WT smokers and 72.20% of the variance in non-exclusive WT smokers (Supplementary Table S2).

### 3.3. Confirmatory Factor Analysis (n = 743)

This eight-item model produced the following indices: χ^2^(15) = 23.317, χ^2^/df = 1.544, CFI = 0.995, TLI = 0.991, RMSEA = 0.0027 and *p*-close = 0.966. Thus, this model displayed good fit. All eight items demonstrated distinctive and salient loadings ranging from 0.48–0.96. The resulting eight-item model was assigned the letter E (referring to Egyptian WT smokers’ data) and was named E8-LWDS to distinguish it from the original 11-item LWDS (Table 2).

### 3.4. Associations between Dependence Risk Using E8-LWDS and Waterpipe Smoking Pattern, Perceived Harm, and Perceived Behavioral Control

E8-LWDS score ranged from 0–24 points. Three levels of dependence risk were identified by applying tertiles to convert the continuous E8-LWDS into categories: score ≤8 was considered low-risk for dependence (*n* = 127, 8.5%), score from >8 to ≤16 was considered moderate-risk (*n* = 880, 59.1%), and score >16 was considered high-risk (*n* = 483, 32.4%). The mean E8-LWDS score was significantly higher among WT smokers who had high-risk for dependence than among those who had low-risk for dependence (Table 3).

In the bivariable analyses, the dependence risk using the E8-LWDS was significantly associated (*p* < 0.001) with all the factors that were hypothesized to be associated with WT dependence, including WTS pattern, worrying about WTS health hazards, and perceived behavioral control, except for three factors: non-exclusive WTS, and WTS perceived harm and WT nicotine content compared to cigarettes (Table 3). For example, participants with high-risk of dependence started WTS at a significantly younger age (17.7 ± 3.1) than low-risk participants (20.5 ± 4.5). Almost all high-risk participants were daily WT smokers compared with one-fifth of low-risk participants. High-risk participants smoked a significantly higher number of hagar (WT portion)/day (7.0 ± 3.7) compared with low-risk participants (3.0 ± 2.0). In addition, 52.8% of the high-risk group spent more than USD 8.6/month on WTS compared to 10.5% of low-risk participants. With respect to perceived harm, worrying about WTS health hazards, was relatively lower in high-risk (62.3%) than in low-risk (92.9%) participants. All the items of perceived behavioral control were significantly associated with participants’ dependence risk. Notably, more than one-half of high-risk participants self-reported themselves to be addicted to WTS (versus 15.7% in moderate-risk and 1.6% in low-risk groups). Moreover, high-risk participants reported significantly lower rates of self-efficacy (19.9%) and fewer quit attempts (7.5%) than moderate-risk (52.0% and 36.2%, respectively) and low-risk (97.6% and 96.1%, respectively) groups (Table 3).

In the generalized linear model, the E8-LWDS score was positively associated with the following: being a daily WT smoker (β = 2.98; 95%CI:2.49, 3.46; *p* < 0.001), self-reported addiction to WTS (β = 1.94; 95%CI:1.56, 2.31; *p* < 0.001), rural residence (β = 1.24; 95%CI:0.65, 1.83; *p* < 0.001), being a skilled worker (β = 1.04; 95%CI:0.63, 1.45; *p* < 0.001), being a non-exclusive WT smoker (β = 0.64; 95%CI:0.35, 0.94; *p* < 0.001), and smoking ≥eight hagars/day (β = 0.51; 95%CI:0.11, 0.92; *p* = 0.014). E8-LWDS score was inversely associated with participants’ perceived ability to quit WTS (β = −1.22; 95%CI:−1.57, −0.87; *p* < 0.001) and previous quit attempts (β = −1.28; 95%CI:−1.67, −0.89; *p* < 0.001) (Table 4).

### 3.5. Group Differentiation

The E8-LWDS total score was able to differentiate between heavy and non-heavy smokers in our sample. We used two different methods to classify WT smokers: (a) frequency of WTS (i.e., waterpipes/week) and (b) intensity (i.e., hagar/day). The median E8-LWDS score in the sample was 14.0 (IQR:11.0–16.0). If this threshold is adopted for dichotomous dependence classification by WTS frequency, 64.4% of heavy smokers (≥7 waterpipes/week) and 15.4% of non-heavy smokers (<7 waterpipes/week) are thus considered WT dependent. By WTS intensity, 72.4% of heavy smokers (≥8 hagars/day) and 45.9% of non-heavy smokers would be considered WT dependent. Similar results were found in exclusive WT and non-exclusive WT smoker groups (Supplementary Table S3).

## 4. Discussion

This is the first study to assess the psychometric properties and factor structure of LWDS-11 in Egypt. Our sample comprised 1490 current WT smokers of various age, gender, and residence. EFA in this population suggested an eight-item scale (E8-LWDS) with a three-factor structure: physical dependence; psychological dependence; and psychological craving. CFA confirmed the E8-LWDS had good model fit.

LWDS factor structure, item subscale assignment, and the items retained varied in the populations previously studied (Supplementary Table S1) [22,23,24,25,26]. LWDS-11 was originally identified via EFA only and yielded a four-factor model: “physiological nicotine dependence”, “negative reinforcement”, “psychological craving”, and “positive reinforcement” [22]. However, CFA was not performed to confirm LWDS-11 construct validity [22]. A Jordanian study identified a three-factor structure using EFA and CFA: “physical dependence,” “relaxation/pleasure,” and “psychosocial aspects” [23]. A Turkish study originally identified a three-factor model using EFA and CFA but a two-factor solution was forced to simplify the model: “physiological dependence” and “psychological dependence” [24]. An Iranian study applied CFA directly to the same four-factor model including all 11 items from the LWDS-11 without a prior EFA [25]. A study in the UK identified a two-factor structure using EFA only: “physiological dependence” and “positive and negative reinforcement” [26].

Despite the variation in LWDS factor structure in different studies/populations, the domain concerned with physical dependence was mostly consistent [22,23,24,25,26]. This was also the case in E8-LWDS except for the item “percent of income you would spend on waterpipe smoking?”, which had factor loading <0.4; thus, it was suppressed by the model. WTS is relatively affordable in Egypt [20]; two-fifths of participants spent <8.6 USD/month on WTS, which may explain why this item was excluded. The Jordanian study noted that this item factor loading was relatively low (0.43) and attributed this to elements other than dependence that affect individuals’ willingness to spend [23].

The remaining domains in LWDS-11 were generally related to psychological aspects and were originally distributed under three dimensions [22]. However, in our study and the Jordanian study [23], they were collapsed under two dimensions. In the Turkish [24] and the UK [26] studies, they were collapsed to one dimension. These domains were labelled differently according to the retained items in each, however, the domain item structure was not consistent across different studies (Supplementary Table S1).

The item “do you smoke waterpipe alone?” was suppressed by E8-LWDS due to factor loading <0.4. It was not discriminative between dependent and non-dependent subjects; 81.2% of participants were daily smokers—a proportion close to that nationally reported (~70%) [31]. Getting to meet friends on daily basis may be inconvenient; this could explain why they generally smoked alone. Factor loading of this item was also relatively low (0.48) in the UK study and it lost its significance when introduced in a multivariable analysis testing risk factors for WT dependence [26]. Furthermore, 53.5% of Egyptian participants smoked at home—similarly to national reports (51.6%) [31], indicating participants’ ability and convenience to smoke alone. Notably, these two items were originally assigned to the “psychological craving” domain in LWDS-11 [22] but they fell under the “physical” domain in the Jordanian [23], the Turkish [24], and the UK [26] studies.

The item “do you smoke to please others?” was excluded from E8-LWDS because it negatively impacted factor reliability, which increased from 0.21 to 0.82 after this item was deleted. This had face validity as dependent subjects would not care to please others because they need WTS to satisfy their addictive habit. This item fell originally under “positive reinforcement” in LWDS-11 and the scale developer called for improving this subscale [22]. This subscale reliability was relatively low in LWDS-11 (0.55) [22] and the UK study (0.50) [26]. The Iranian study attributed the low reliability (0.45) of the subscale to this item as WTS is practiced for pleasure of the smoker not others in the Iranian culture [25]. The Turkish study also excluded this item [24]. The UK study recommended deleting this item because its corrected item-total correlation was very low (0.14) [26]. Different populations have different psychological characteristics, but a generic scale would be expected to show some consistency in the psychological domain as that in the physical domain. In addition, LWDS total reliability and variance explained varied in different studies (Supplementary Table S1).

CFA confirmed E8-LWDS had good model fit. This was also the case for LWDS-10J, [23] LWDS-TR, [24] and LWDS in the Iranian study [25]. The fact that all the different studies showed good model fit despite different factor structures, implies that each adjusted scale is suitable for the population in which it was studied, but it may not be valid across other populations.

Adding other items that significantly discriminate between dependent and non-dependent subjects may increase E8-LWDS total scale reliability. This is in line with the findings of Alam et al., 2020 in their study on schoolchildren in Beirut, Lebanon [12]. Perceived behavioral control was notably different across groups of WT dependence in our study. For instance, half of high-risk participants self-reported addiction to WTS. This item alone could be useful in screening high-risk individuals for WT dependence and may facilitate their linkage to cessation services. In addition, only one-fifth of the high-risk group thought they had the ability to quit WTS whenever they wanted, and less than a tenth reported previous quit attempts. This reflects their low self-efficacy as would be expected in high-risk groups. The opposite was found in the low-risk group. Previous studies also reported an inverse relationship between perceived self-efficacy and perceived addiction to WTS [11,20]. Noteworthy, 96.3% of the high-risk group reported daily WTS compared to 21.3% of the low-risk group, reflecting the great need of these highly dependent subjects for a daily source of nicotine. In addition, these items were independently associated with E8-LWDS score in multivariable analysis.

E8-LWDS identified one-third of participants as high-risk for WT dependence, a proportion similar to that identified by LWDS-10J [23]. E8-LWDS demonstrated good convergent construct validity with factors that were hypothesized to be associated with WT dependence, whether the scale was represented in tertiles or as a continuous variable, suggesting these results were robust. Daily WTS smoking, self-reported addiction, higher frequency/intensity of use, perceived ability to quit, and previous quit attempts were also found to be associated with WT dependence in previous studies [10,11,12,13,14,15,16,17,18,19,20,22,23,24,25,26]. E8-LWDS was able to discriminate between heavy and non-heavy WT smokers using WTS frequency (waterpipes/week) and intensity (hagar/day) as in other studies [14,22]. This discriminatory ability was also evident in exclusive and non-exclusive WTS groups, indicating the discriminant validity of the E8-LWDS across various smoker groups and frequency/intensity classifications.

### Strengths and Limitations

This study included a large population of current WT smokers recruited via purposive quota sampling to include nationally representative proportions of age, gender, rural/urban residence and WTS status. Although the sampling method was non-random, WTS characteristics exhibited by this sample were comparative to the national WTS context (proportion of daily WTS and home WTS). The scale was not previously assessed on rural WT smokers [22,23,24,25,26]. Most Egyptian WT smokers are rurally located [32]; rural residence was independently associated with E8-LWDS score in multivariable analysis. This sample structure allowed a practical assessment of the LWDS.

It was important to assess the model among exclusive and non-exclusive WT smokers separately because multiple nicotine/tobacco product use is common [7]. Almost half of this sample were non-exclusive WT smokers. In addition, non-exclusive WTS was independently associated with E8-LWDS score in multivariable analysis, implying that dependence does not merely arise from using the waterpipe itself or its social context, but also from an addictive substance in WT [35,36]. No other study assessed their model in exclusive and non-exclusive WT smokers [22,23,24,25,26]. The eight-item structure was stable in both analyses.

Participants’ self-reported measures were not verified by laboratory tests and may be subject to recall bias. However, self-reports of smoking behaviour have been argued to be valid [37]. Further, self-reporting of addiction is an important milestone of behavioural change. As in all studies that assessed the LWDS so far, this study design was cross-sectional; further in-depth studies could support the development of a more robust scale for assessing WT dependence.

## 5. Conclusions

EFA in this population of 1490 Egyptian current waterpipe smokers suggested an eight-item scale (E8-LWDS) with a three-factor structure: physical dependence; psychological dependence; and psychological craving. The model was stable in exclusive and non-exclusive WT smokers. CFA confirmed E8-LWDS had good model fit. E8-LWDS was similar to the original LWDS-11 scale in the physical and psychological craving domains. It displayed good convergent construct validity with factors conceptually expected to be associated with WT dependence. Including items related to perceived behavioral control, such as self-reported addiction, self-efficacy, and previous quit attempts could improve scale reliability. E8-LWDS demonstrated adequate reliability and validity and may assist practitioners in identifying WT dependent subjects in clinical settings to facilitate their linkage to cessation services. Scales measuring WT dependence still need further research. Further in-depth studies among different populations and settings could support the development of a more robust scale for assessing WT dependence.

## Figures and Tables

**Table 1:** Current waterpipe tobacco smokers’ background characteristics, *n* = 1490.

Background Characteristics	Total
	*n* (%) ^a^
**Age group**	
18–24 years	535 (35.9)
≥25 years	955 (64.1)
**Gender**	
Male	1361 (91.3)
Female	129 (8.7)
**Residence**	
Rural	883 (59.3)
Urban	607 (40.7)
**Education level**	
No schooling	75 (5.0)
Primary	118 (7.9)
Preparatory	139 (9.3)
Secondary	554 (37.2)
Vocational/university	604 (40.5)
**Occupation**	
Professional	18 (12.7)
Technicians and associate professionals	134 (9.0)
Skilled	699 (46.9)
Elementary occupations	157 (10.5)
Student or unemployed	311 (20.9)
**Marital status**	
Unmarried	527 (35.4)
Married	963 (64.6)
**Household SHS exposure**	
No	481 (32.3)
Yes	1009 (67.7)
**Waterpipe tobacco smoking status**	
Exclusive	777 (52.1)
Non-exclusive	713 (47.9)

**^a^** Due to rounding, sums of relative frequency presented may not add up to exactly 100%; SHS, secondhand smoke.

**Table 2:** Exploratory and confirmatory factor analysis, factor loadings, and structure of the E8-LWDS among current waterpipe tobacco smokers.

			E8-LWDS EFA Factor Loadings and Subscale Assignment (*n* = 747)					E8-LWDS CFA Factor Loadings and Subscale Assignment (*n* = 743)			
	Items Retained from the Original LWDS-11	Original LWDS-11 Subscale Assignment	1 *	2 *	3 *	Corrected Item-Total Correlation	Cronbach’s Alpha if Item Deleted		1 *	2 *	3 *
**1**	Number of times you could stop waterpipe for >7 days	1	0.824			0.418	0.576	Standardized regression weights	0.705		
**3**	Number of days you could spend without waterpipe	1	0.913			0.443	0.575		0.957		
**4**	Number of waterpipes you usually smoke per week	1	0.860			0.298	0.613		0.811		
**5**	Do you smoke waterpipe to relax your nerves	2		0.761		0.303	0.613			0.475	
**6**	Do you smoke waterpipe to improve your morale	2		0.802		0.417	0.576			0.729	
**7**	Do you smoke waterpipe when you are seriously ill	3			0.907	0.219	0.629				0.821
**9**	Are you ready not to eat in exchange for a waterpipe	3			0.898	0.270	0.618				0.764
**10**	Do you smoke waterpipe for pleasure	4		0.734		0.274	0.619			0.552	
	Eigen values		2.381	1.872	1.492			χ^2^/df	23.317	15	1.554
	% Variance explained by factor		29.762	23.398	18.646			TLI	0.991		
	Total variance explained by model		71.807					CFI	0.995		
	Cronbach’s alpha for factors		0.823	0.654	0.785			RMSEA	0.027		
	Cronbach’s alpha for total scale		0.635					PCLOSE	0.966		

EFA, exploratory factor analysis; CFA, confirmatory factor analysis; TLI, Tucker–Lewis index; CFI, comparative fit index; RMSEA, root mean square error approximation; PCLOSE, *p*-value for a close fit. * Factor 1: physical dependence, Factor 2: psychological dependence, and Factor 3: psychological craving.

**Table 3:** Associations between risk of dependence using E8-LWDS and current waterpipe tobacco smokers’ pattern, perceived harm, and perceived behavioral control.

	Total	Risk of Dependance Using E8-LWDS				
		Low	Moderate	High		
	*N* = 1490	*n* = 127	*n* = 880	*n* = 483	Statistic	*p*-Value
E8-LWDS score, mean (SD)	13.8 ± 3.8	6.3 ± 1.8	12.6 ± 1.9	17.8 ± 1.9	F = 2311.156	<0.001
Hagar smoked per day (number), mean (SD)	5.8 ± 3.5	3.0 ± 2.0	5.5 ± 3.3	7.0 ± 3.7	F = 77.486	<0.001
Age at WTS initiation (years), mean (SD)	18.3 ± 3.5	20.5 ± 4.5	18.4 ± 3.5	17.7 ± 3.1	F = 34.181	<0.001
	*n* (%)	*n* (%)	*n* (%)	*n* (%)	χ^2^	
Non-exclusive WTS, yes	713 (47.9)	71 (55.9)	416 (47.3)	226 (46.8)	1.799	0.180
Frequency of WTS, daily	1210 (81.2)	27 (21.3)	718 (81.6)	465 (96.3)	276.574	<0.001
Smokes waterpipe usually alone, yes	1041 (69.9)	65 (51.2)	625 (71.0)	351 (72.7)	12.565	<0.001
Usual place of WTS, home	797 (53.5)	33 (26.0)	475 (54.0)	289 (59.8)	32.905	<0.001
Usual source of waterpipe tobacco, self-purchase	817 (54.8)	38 (29.9)	487 (55.3)	292 (60.5)	26.573	<0.001
Monthly spending on WTS,^a^ ≥ 150 EGP (USD 8.6)	573 (38.5)	13 (10.5)	305 (34.7)	255 (52.8)	87.136	<0.001
Compared to cigarettes, WTS is more harmful	820 (55.0)	64 (50.4)	490 (55.7)	266 (55.1)	0.284	0.594
Compared to cigarettes, WTS contains more nicotine	245 (16.4)	22 (17.3)	144 (16.4)	79 (16.4)	0.033	0.856
Worry about WTS health hazards, yes	1096 (73.6)	118 (92.9)	677 (76.9)	301 (62.3)	60.871	<0.001
Self-reported addiction to WTS, yes	384 (25.8)	2 (1.6)	138 (15.7)	244 (50.5)	224.660	<0.001
Perceived ability to quit WTS, yes	678 (45.5)	124 (97.6)	458 (52.0)	96 (19.9)	277.111	<0.001
Previous quit attempts,^b^ yes	476 (32.0)	122 (96.1)	318 (36.2)	36 (7.5)	349.487	<0.001

WTS, waterpipe tobacco smoking. **^a^** Three observations missing; **^b^** six observations missing.

**Table 4:** Generalized linear model for associations of E8-LWDS score with background characteristics, WTS pattern, perceived harm, and perceived behavioral control of waterpipe tobacco smoking.

	E8-LWDS Score					
	β	SE	95% CI		Wald Chi-Square	*p*-Value
**Background Characteristics**						
Age (≥25 years)	−0.123	0.2323	−0.578	0.333	0.278	0.598
Gender (male)	0.594	0.3073	−0.008	1.197	3.739	0.053
Residence (rural)	1.235	0.3017	0.644	1.827	16.761	<0.001
Education (lower)	−0.094	0.2324	−0.550	0.362	0.164	0.686
Occupation (skilled)	1.037	0.2095	0.627	1.448	24.531	<0.001
Marital Status (married)	−0.067	0.2360	−0.529	0.396	0.080	0.778
Exposure to secondhand smoke (yes)	0.078	0.1955	−0.305	0.461	0.161	0.689
Non-exclusive WTS (yes)	0.641	0.1510	0.345	0.937	18.003	<0.001
**WTS pattern**						
Age at WTS initiation (≥18 years)	0.216	0.1601	−0.098	0.530	1.824	0.177
Frequency of WTS (daily)	2.976	0.2449	2.496	3.456	147.740	<0.001
Hagar per day (≥8)	0.513	0.2083	0.105	0.921	6.061	0.014
Smokes waterpipe usually alone (yes)	0.210	0.1826	−0.148	0.568	1.324	0.250
Usual place of WTS (home)	0.085	0.2944	−0.492	0.662	0.083	0.773
Usual source of waterpipe tobacco (self-purchase)	−0.052	0.2883	−0.617	0.513	0.032	0.857
Monthly spending on WTS (≥150 EGP (USD 8.6))	0.208	0.1879	−0.161	0.576	1.222	0.269
**Harm perception**						
Compared to cigarettes (WTS is more harmful)	−0.035	0.2034	−0.433	0.364	0.029	0.865
Compared to cigarettes (WTS contains more nicotine)	0.183	0.2123	−0.233	0.599	0.741	0.389
Worry about WTS health hazards (yes)	−0.281	0.1933	−0.659	0.098	2.109	0.146
**Perceived behavioral control**						
Self-reported addiction to WTS (yes)	1.936	0.1931	1.557	2.314	100.497	<0.001
Perceived ability to quit WTS (yes)	−1.216	0.1786	−1.566	−0.866	46.368	<0.001
Previous quit attempts (yes)	−1.281	0.1998	−1.673	−0.890	41.108	<0.001

## Data Availability

All relevant data are within the manuscript and its supplementary material.

## Supplementary Materials

The following are available online at https://www.mdpi.com/article/10.3390/ijerph18136741/s1, Table S1: The LWDS factor structure in different studies, Table S2: Exploratory factor analysis, factor loadings, and structure of the E8-LWDS among exclusive and non-exclusive current waterpipe smokers, Table S3: Differentiation between exclusive and non-exclusive waterpipe tobacco smokers (median test).
