# Supplementary material for: E8-LWDS: Factorial Structure and Psychometric Properties of the Lebanese Waterpipe Dependence Scale-11 in 1490 Egyptian Waterpipe Tobacco Smokers—A Critical Approach
Source: Int J Environ Res Public Health. 2021 Jun 23;18(13):6741. doi: 10.3390/ijerph18136741 (PMC8269008; doi:10.3390/ijerph18136741)
Supplement: Supplementary file 1 [file ijerph-18-06741-s001.zip › ijerph-1207926-supplementary.pdf]

## Supplementary Table S1. The LWDS factor structure in different studies

| Country where study was conducted | Lebanon* | Iran | Egypt | Turkey | Jordan | The United Kingdom |
|---|---|---|---|---|---|---|
| **Acronym** | **LWDS-11** | **Not available** | **E8-LWDS** | **LWDS-TR** | **LWDS-10J** | **Not available** |
| **Analysis methods** | EFA | CFA | EFA & CFA | EFA & CFA | EFA & CFA | EFA |
| **Sample size** | 291 (103/188) | 465 | 1490 | 401 | 2577 | 180 |
| **Sample description** | Current exclusive waterpipe smokers (>1 waterpipe/2 weeks) Males & females (33.0%/39.9%) Mean age 29.7/36.2 | Current waterpipe smokers (any WTS in past month) Males & females (73.7%) Age range 15-54 | Current waterpipe smokers (any WTS in past-30 days) Non-exclusive WTS included Males & females (8.7%) Age range 18-87 | Current waterpipe smokers (≥1 waterpipe/2 weeks) Dual waterpipe & cigarette smokers included Males & females (28.2%) Age range 18-62 | Ever waterpipe smoker university students Males & females (56%) Mean age 21.2 | Waterpipe smokers (any WTS in past year) Males only Mean age 29.5 |
| **Place of conduction** | Urban Beirut cafés | Urban Tehran | Rural Menoufia & urban Cairo cafes, households, workplaces, & universities | Urban Ankara waterpipe serving establishments & social media | Public & private universities | Urban London cafés |
| **Sampling method** | Convenience/random digital dialing | Random | Purposive quota representing national proportions of age, gender, residence, & WTS | Non-random | Random | Non-random |
| **Number of factors extracted** | **4** | **4** | **3** | **2** | **3** | **2** |
| **Number of items included** | **11** | **11** | **8** | **9** | **10** | **10** |
| 1. Number of times you could stop waterpipe for >7 days? | Physiological nicotine dependence | Physiological nicotine dependence | Physical dependence | Physiological dependence | Physical dependence | Physiological dependence |
| 2. Percent of income you would spend on waterpipe smoking? | Physiological nicotine dependence | Physiological nicotine dependence | Not included | Physiological dependence | Physical dependence | Physiological dependence |
| 3. Number of days you could spend without waterpipe? | Physiological nicotine dependence | Physiological nicotine dependence | Physical dependence | Physiological dependence | Physical dependence | Physiological dependence |
| 4. Number of waterpipes you usually smoke per week? | Physiological nicotine dependence | Physiological nicotine dependence | Physical dependence | Physiological dependence | Physical dependence | Physiological dependence |
| 5. Do you smoke waterpipe to relax your nerves? | Negative reinforcement | Negative reinforcement | Psychological dependence | Psychological dependence | Relaxation/ pleasure | Post/neg reinforcement |
| 6. Do you smoke waterpipe to improve your morale? | Negative reinforcement | Negative reinforcement | Psychological dependence | Psychological dependence | Psychosocial | Post/neg reinforcement |
| 7. Do you smoke waterpipe when you are seriously ill? | Psychological craving | Psychological craving | Psychological craving | Physiological dependence | Physical dependence | Physiological dependence |
| 8. Do you smoke waterpipe alone? | Psychological craving | Psychological craving | Not included | Physiological dependence | Physical dependence | Physiological dependence |
| 9. Are you ready not to eat in exchange for a waterpipe? | Psychological craving | Psychological craving | Psychological craving | Physiological dependence | Not included | Not included |
| 10. Do you smoke waterpipe for pleasure? | Positive reinforcement | Positive reinforcement | Psychological Dependence | Not included | Relaxation/ pleasure | Post/neg reinforcement |
| 11. Do you smoke to please others (conviviality)? | Positive reinforcement | Positive reinforcement | Not included | Not included | Psychosocial | Post/neg reinforcement |
| **Total scale reliability** | 0.83/0.70 | Not applicable | 0.64 | 0.8 | 0.77 | 0.74 |
| **Subscale reliability (min, max)** | 0.55/0.42, 0.88/0.69 | Not applicable | 0.65, 0.82 | 0.81, 0.82 | 0.67, 0.75 | 0.50, 0.80 |
| **Total % of variance explained** | 68.97/not mentioned | Not applicable | 71.8 | 59 | Not mentioned | 47.8 |

**EFA, Exploratory factor analysis; CFA, Confirmatory factor analysis; WTS, waterpipe tobacco smoking**

*Sample 1/Sample 3. (%) represent proportion of females in the study sample.

Different cell shade by the same colour (i.e. light and dark blue; light and dark yellow; light and dark gray) indicates different factor labelling or different item subscale assignment.

**Supplementary Table S2. Exploratory factor analysis, factor loadings, and structure of the E8-LWDS among exclusive and non-exclusive current waterpipe smokers**

| | | | E8-LWDS factor loadings and subscale assignment in Exclusive WTS (n=777) | | | | | E8-LWDS factor loadings and subscale assignment in Non-exclusive WTS (n=713) | | | | |
|---|---|---|---|---|---|---|---|---|---|---|---|---|
| | **Items retained from the original LWDS-11** | **LWDS-11 original subscale assignment** | **1*** | **2*** | **3*** | **Corrected Item-Total Correlation** | **Cronbach's Alpha if Item Deleted** | **1*** | **2*** | **3*** | **Corrected Item-Total Correlation** | **Cronbach's Alpha if Item Deleted** |
| 1 | **Number of times you could stop waterpipe for >7 days** | 1 | **0.846** | | | 0.412 | 0.578 | **0.814** | | | 0.411 | 0.532 |
| 3 | **Number of days you could spend without waterpipe** | 1 | **0.902** | | | 0.419 | 0.582 | **0.926** | | | 0.423 | 0.531 |
| 4 | **Number of water pipes you usually smoke per week** | 1 | **0.854** | | | 0.338 | 0.606 | **0.885** | | | 0.311 | 0.567 |
| 5 | **Do you smoke waterpipe to relax your nerves** | 2 | | **0.741** | | 0.358 | 0.596 | | | **0.710** | 0.231 | 0.592 |
| 6 | **Do you smoke waterpipe to improve your morale** | 2 | | **0.818** | | 0.386 | 0.587 | | | **0.787** | 0.392 | 0.536 |
| 7 | **Do you smoke waterpipe when you are seriously ill** | 3 | | | **0.882** | 0.113 | 0.651 | | **0.924** | | 0.274 | 0.577 |
| 9 | **Are you ready not to eat in exchange for a waterpipe** | 3 | | | **0.859** | 0.27 | 0.618 | | **0.927** | | 0.268 | 0.577 |
| 10 | **Do you smoke waterpipe for pleasure** | 4 | | **0.768** | | 0.354 | 0.596 | | | **0.713** | 0.128 | 0.619 |
| | **Eigen values** | | 2.428 | 1.790 | 1.445 | | | 2.408 | 1.891 | 1.475 | | |
| | **% Variance explained by factor** | | 30.352 | 22.377 | 18.065 | | | 30.106 | 23.641 | 18.440 | | |
| | **Total Variance explained by model** | | 70.794 | | | | | 72.187 | | | | |
| | **Cronbach's alpha for factors** | | 0.823 | 0.676 | 0.692 | | | 0.846 | 0.833 | 0.585 | | |
| | **Cronbach's alpha for total scale** | | 0.635 | | | | | 0.600 | | | | |

**WTS, waterpipe tobacco smoking; Non-exclusive WTS, waterpipe tobacco smoking and/or cigarette smoking and/or electronic nicotine delivery systems use**

*** Factor 1: physical dependence, Factor 2: psychological dependence, and Factor 3: psychological craving.**

**Supplementary Table S3. Differentiation between exclusive and non-exclusive waterpipe tobacco smokers (median test)**

| | Total sample (N=1490) | | Exclusive WTS (N=777) | | Non-exclusive WTS (N=713) | |
|---|---|---|---|---|---|---|
| | **Non-heavy smoker** | **Heavy smoker** | **Non-heavy smoker** | **Heavy smoker** | **Non-heavy smoker** | **Heavy smoker** |
| **Hagar (tobacco portion) smoked per day (number)** | **<8** | **≥8** | **<8** | **≥8** | **<8** | **≥8** |
| | **n=964** | **n=526** | **n=476** | **n=301** | **n=488** | **n=223** |
| E8-LWDS mean (SD)[a] | 13.0 (4.0) | 15.2 (2.7) | 13.2 (3.9) | 15.4 (2.8) | 12.8 (4.1) | 15.0 (2.6) |
| E8-LWDS median (IQR) | 13.0 (10.0-15.0) | 15.0 (13.0-17.0) | 13.0 (11.0-15.0) | 15.0 (13.0-18.0) | 13.0 (10.0-16.0) | 15.0 (13.0-17.0) |
| Percentage above median hagar/day[b]* | 35.2 | 61.8 | 35.1 | 62.5 | 35.2 | 61.4 |
| Percentage above E8-LWDS score 14[c]* | 45.9 | 72.4 | 48.3 | 72.4 | 43.4 | 72.4 |
| | **Non-heavy smoker** | **Heavy smoker** | **Non-heavy smoker** | **Heavy smoker** | **Non-heavy smoker** | **Heavy smoker** |
| **Waterpipes smoked per week (number)** | **<7** | **≥7** | **<7** | **≥7** | **<7** | **≥7** |
| | **n=279** | **n=1211** | **n=101** | **n=676** | **n=178** | **n=535** |
| E8-LWDS mean (SD)[a] | 9.9 (3.8) | 14.7 (3.1) | 9.4 (4.0) | 14.7 (3.1) | 10.1 (3.7) | 14.6 (3.1) |
| E8-LWDS median (IQR) | 10.0 (7.0-12.0) | 15.0 (16.0-17.0) | 9.0 (7.0-12.5) | 15.0 (13.0-17.0) | 10.0 (8.0-12.0) | 15.0 (12.0-17.0) |
| Percentage above median waterpipes/week[b]* | 11.1 | 52.3 | 11.9 | 50.7 | 10.7 | 54.2 |
| Percentage above E8-LWDS score 14[c]* | 15.4 | 64.4 | 14.9 | 64.1 | 15.7 | 64.9 |

**WTS, waterpipe tobacco smoking; Non-exclusive WT use, waterpipe tobacco smoking and/or cigarette smoking and/or electronic nicotine delivery systems use**

[a] Independent samples T-test

[b] Median nonparametric test

[c] Chi-squared test

*All p-values were <0.001
